# Altered Expression of Peroxiredoxins in Mouse Model of Progressive Myoclonus Epilepsy upon LPS-Induced Neuroinflammation

**DOI:** 10.3390/antiox10030357

**Published:** 2021-02-27

**Authors:** Mojca Trstenjak Prebanda, Petra Matjan-Štefin, Boris Turk, Nataša Kopitar-Jerala

**Affiliations:** 1 Department of Biochemistry, Molecular and Structural Biology, Jožef Stefan Institute, SI-1000 Ljubljana, Slovenia; mojca.prebanda@ijs.si (M.T.P.); petra.matjan-stefin@ijs.si (P.M.-Š.); boris.turk@ijs.si (B.T.); 2 International Postgraduate School Jožef Stefan, Jamova 39, SI-1000 Ljubljana, Slovenia; 3 Faculty of Chemistry and Chemical Technology, University of Ljubljana, Večna pot 113, SI-1000 Ljubljana, Slovenia; 4 Institute of Regenerative Medicine, I.M. Sechenov First Moscow State Medical University, Bol’shaya Pirogovskaya Ulitsa, 19c1, 119146 Moscow, Russia

**Keywords:** brain, cystatin, EPM1, neuro inflammation, LPS-induced oxidative stress, peroxiredoxin, thioredoxin, thioredoxin reductase

## Abstract

Stefin B (cystatin B) is an inhibitor of endo-lysosomal cysteine cathepsin, and the loss-of-function mutations in the stefin B gene were reported in patients with Unverricht–Lundborg disease (EPM1), a form of progressive myoclonus epilepsy. Stefin B-deficient mice, a mouse model of the disease, display key features of EPM1, including myoclonic seizures. Although the underlying mechanism is not yet completely clear, it was reported that the impaired redox homeostasis and inflammation in the brain contribute to the progression of the disease. In the present study, we investigated if lipopolysaccharide (LPS)-triggered neuroinflammation affected the protein levels of redox-sensitive proteins: thioredoxin (Trx1), thioredoxin reductase (TrxR), peroxiredoxins (Prxs) in brain and cerebella of stefin B-deficient mice. LPS challenge was found to result in a marked elevation of Trx1 and TrxR in the brain and cerebella of stefin B deficient mice, while Prx1 was upregulated only in cerebella after LPS challenge. Mitochondrial peroxiredoxin 3 (Prx3), was upregulated also in the cerebellar tissue lysates prepared from unchallenged stefin B deficient mice, while after LPS challenge Prx3 was upregulated in stefin B deficient brain and cerebella. Our results imply the role of oxidative stress in the progression of the disease.

## 1. Introduction

Unverricht–Lundbordg disease (EPM1, OMIM 254800) is a type of progressive myoclonus epilepsy, which is inherited autosomal recessively. The onset of the disease is in childhood and progress of the disease is associated with neurological symptoms such as myoclonus, epilepsy, and ataxia [1]. The underlying cause is mutations in the gene encoding stefin B (cystatin B), an intracellular endogenous inhibitor of cysteine cathepsins [2,3]. In patients, the most frequent mutation is an expansion of a dodecamere repeat in the 5′-untranslated region of the stefin B gene, which results in lower expression of stefin B protein, while frameshift mutations and deletions are less common in EPM1 patients [4]. The underlying mechanism is not yet clear, but it was reported that increased reactive oxygen species (ROS) and inflammation contribute to the disease [5,6]. In the cells, stefin B is found in cytosol, as well as in the nucleus and mitochondria [7,8,9,10]. We reported that stefin B interacted with nucleosomes, specifically with histones H2A.Z, H2B, and H3 and cathepsin L in the nucleus [8]. A recent study confirmed that stefin B acts as an endogenous modulator of histone H3 tail trimming by the inhibition of cathepsins B and L in the nuclei of mouse neural progenitor cells [11]. It was demonstrated that stefin B expression is higher upon oxidative stress and macrophage activation [5,12]. Stefin B-deficient mice, a mouse model of EPM1, recapitulate key features of the human disease, including myoclonic seizures and progressive brain degeneration [13,14]. Prior to the onset of seizures and apoptosis in cerebellar neurons, mice had activation of microglia cells and neuroinflammation [15,16]. In our previous work, we reported that stefin B-deficient mice were significantly more sensitive to LPS induced sepsis due to increased caspase-11 gene expression, enhanced inflammasome activation, and increased secretion of pro-inflammatory cytokines [9]. Stefin B-deficient bone marrow-derived macrophages secreted higher amounts of proinflamamtory cytokines interleukin 1β (IL-1β), IL-18, TNFά, and less anti-inflammatory IL-10 [9,12]. LPS challenge is frequently used to study neuroinflammation-associated diseases in mice [17]. Housewearat et al. examined the role of individual cathepsins in the progression of EPM1, by crossing stefin B deficient mice with cathepsin B-, L-, and S-deficient mice and examined pathology of the disease in these double deficient mouse models [18]. Interestingly, lack of cathepsins L or S in stefin B-deficient mice did not ameliorate the disease, but the removal of cathepsin B resulted in a significant down regulation of apoptosis in crebellum. However, ataxia and myoclonus were not diminished, indicating that other factors, not only cathepsins contribute to pathology of EPMI [18]. Since antioxidant N-acetylcysteine (NAC) alleviated seizures and myoclonus in EPM1 patients [19,20], additional factors that participate in a disease progression could be related to oxidative stress regulation. However, the importance of peroxiredoxin expression in neuronal degeneration in EPM1 has remained unexplored.

The thioredoxin system (Trx), which is one of the major cellular systems, suppresses oxidative stress in the cells through its disulfide reductase activity, is composed of Trx, thioredoxin reductase (TrxR), and NADPH [21]. Trx is localized in the cell cytoplasm of different tissues and it protects cells from ROS by reducing the disulfides in the proteins [22]. It is regenerated by the action of TrxR [23]. Peroxiredoxins (Prxs) area family of antioxidant enzymes that function in regulating peroxide levels in cells and tissue [24]. Most of them are active as homodimers and use redox-active cysteines to reduce peroxides. Prxs family has three subgroups: 2-Cys Prx which contain an additional conserved cysteine in the C-terminal region, while the peroxiredoxins of two other groups (atypical 2-Cys, and 1-Cys) do not have it [25]. N-terminal cysteine of typical 2-Cys Prxs (Prx1, – 4), is oxidized by hydrogen peroxide to a cysteine-sulfenic acid that then reacts with the C-terminal cysteine of another subunit and forms an intermolecular disulfide bond. Trx/TrxR system could reduce this disulfide bond and this way terminates the catalytic cycle. While Prx1 and Prx2 are found in the cytosol, Prx3 is strictly mitochondrial where it protects mitochondria from ROS [24,25,26]. Sulfiredoxin (Srx) is a sulfinic acid reductase and it catalyzes the Cys-sulfinic acid derivatives of Prxs to reduced forms in an ATP-dependent reaction [27,28,29,30].

During the past ten years, several studies reported the protective role of Prx in LPS induced sepsis [31,32,33]. In addition, Prx3 deficient mice were reported to be more sensitive to LPS induced sepsis [34]

Although the role of oxidative stress in stefin B deficient mouse model was reported more than a decade ago [5,35], the role of antioxidant enzymes Trxs and Prxs in LPS induced oxidative stress and neuroinflamamtion in the mouse model of EPM1 is poorly understood. In our previous study on spleens and macrophages of stefin B deficient mice, we determined a marked increase of mitochondrial Prx3, Srx and superoxide dismutase 2 in stefin B deficient cells after LPS stimulation [36].

The objective of this study was to understand the fundamental processes of the regulation of oxidative stress in the tissue where the disease linked to stefin B deficiency is occurring. Specifically, we focused on redox-sensitive protein Prx1–3 Trx and TrxR protein expression in the brain (without cerebella) and cerebella of LPS treated WT and stefin B deficent animals. LPS challenge was found to result in a marked elevation of Trx1 and TrxR in the brain and cerebella of stefin B-deficient mice and Prx1 in cerebella after LPS challenge. Mitochondrial Prx3 was upregulated also in the cerebellar cell lysates prepared from unchallenged stefin B deficient mice, while after LPS challenge Prx3 was upregulated in stefin B deficient brain and cerebella. Our results imply the role of mitochondrial stress in the LPS induced neuroinflammation.

## 2. Materials and Methods

### 2.1. Reagents

For the in vitro experiments, LPS from E. coli LPS (*E. coli*, Sigma Aldrich, St Louis, MO, USA) was used. Most of the antibodies used for Western blots were from Abcam (Abcam, Cambridge, UK) anti Prx pathway (Trx1, TrxR, and Prx1) cocktail (ab184868), anti peroxiredoxin 2 (ab109367), anti peroxiredoxin 3 (ab73349). Antibodies anti β-actin were from Sigma (A1978) (Sigma Aldrich, St Louis, MO, USA).

### 2.2. Animals

Stefin B (cystatin B)-deficient mice were generated as described before [35]. Mice (8–12 weeks of age) used in this study, fully backcrossed to FVB/N background, were wild type (WT) and stefin B-deficient (KO). All animal studies, maintenance and breeding, were conducted in accordance with the Administration of the Republic of Slovenia for food safety, veterinary and plant protection. Procedures for animal care and experiments were in accordance with the “Guide for the Care and Use in Laboratory Animals,” approved by the Veterinary Administration of the Republic of Slovenia and the government Ethical Committee (Ethical protocol code: 34401-49/2014/7).

### 2.3. Stimulations and Systemic LPS Challenge In Vivo

For the whole brain and cerebella whole tissue lysate preparations, WT and stefin B KO mice were intraperitoneally injected with LPS at 3 mg/kg (*E. coli* 055:B5, Sigma) (Sigma Aldrich, St Louis, MO, USA). Experiments were conducted on male and female mice, at least 3 animals for condition we reused in experiments. Anasthetics were used according to protocols, were a mixture of ketamine/xylazine/acepromazine. Animals were sacrificed 4 h after LPS injection.

### 2.4. Cell Lysate Preparation and Western Blot Analysis

Mouse tissue lysate was prepared by homogenization in the Nonidet P-40 lysis buffer (20 mM Tris–HCl (pH 7.4), 150 mM NaCl, 1 mM EDTA, 1% Nonidet P-40, 10% glycerol, 1 mM sodium orthovanadate, 10 mM NaF, 10 mM β-glycerophosphate), supplemented with the complete protease inhibitor cocktail (Sigma) and the phosphatase inhibitor cocktail (Sigma Aldrich, St. Louis, MO, USA). After cell and tissue debris were removed, protein concentration was determined using Bradford reagent (Bio-Rad). The lysates were separated on 12.5% SDS-polyacrylamide gels, and Western blots were performed, as described previously [9]. Specific bands were developed by enhanced chemiluminescence (ECL) (Amersham Biosciences, Amersham, UK), according to the manufacturer’s instructions. The signals were quantified by densitometry analysis using ImageJ software (http://rsb.info.nih.gov/ij/index.html accessed on 21 November 2019), according to the instructions, as described: https://www.unige.ch/medecine/bioimaging/files/2014/1208/6025/GelAnalysis accessed on 21 November 2019).

### 2.5. Statistical Analysis

Statistical significance of the results was calculated using unpaired Student’s *t*-test, assuming unequal variances. The results are expressed as the mean ± standard error of the mean. When *p*-values were 0.05 or less, differences were considered statistically significant.

## 3. Results

### 3.1. Tiroxiredoxin (Trx) and TrxR Are Upregulated in LPS Induced Neuroinflamamtion in Brain Lysates from Stefin B-defficient Mice

**Figure 1:**
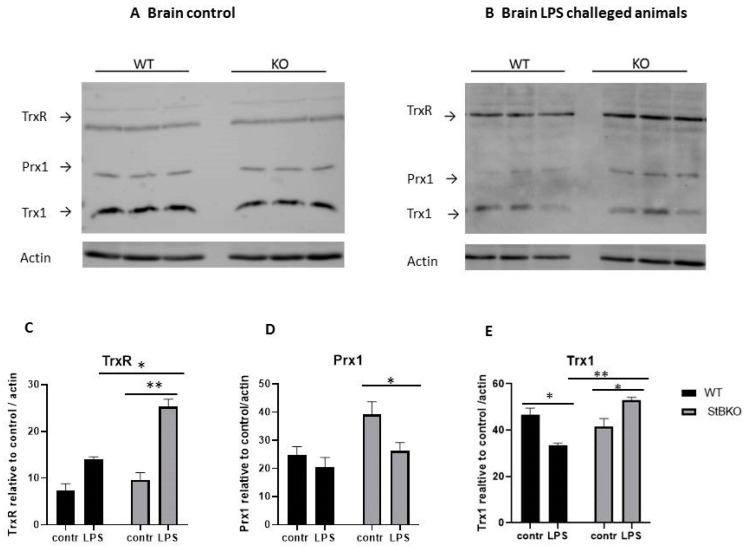
Thioredoxin reductase (TrxR), peroxiredoxin 1 (Prx1) and thioredoxin 1 (Trx1) protein levels in brain of stefin B-deficient (KO) and control (WT) mice (n = 6) after LPS challenge. Age-matched control FVB/N mice (WT) and stefin B-deficient (KO) were left untreated or were injected with LPS (3 mg/kg body weight). 4 h after LPS challenge animals were sacrificed. Brain tissue lysates of control mice (**A**) or LPS injected animals (**B**) were analyzed by Western blots with specific antibodies. Anti β-actin antibodies were used as a loading controls. The levels of TrxR (**C**), Prx1 (**D**) and Trx1(**E**) in brain lysates from wild type (WT) and stefin B-deficient (KO) mice in comparison to β-actin controls were quantified by ImageJ. Statistically significant differences vs. control group, * *p* < 0.05. ** *p* ≤ 0.01.

In our previous study, we examined the expression of TrxR, Prx1, and Trx in LPS stimulated stefin B-deficient macrophages and spleens. Cytosolic Prx1 regulates hydrogen signaling through its peroxidase activity [25]. In the present study, we examined if the lack of stefin B influence Prx1 protein levels in WT and stefin B-deficient brain (without cerebella) after systemic LPS stimulation. In addition, we examined also the other proteins involved in the Prx-reduction and regeneration: Trx1 and TrxR. Trx1 is found in glial and neuronal cells and control redox signaling. The Tiroxiredoxin (Trx) system, coupled with Prx1/2, regulates redox signaling in the cytosol and nucleus. In the brain tissue from control animals, we did not detect any significant differences between the genotypes in the protein levels of Prx1, Trx1, and TrxR (Figure 1A,C,D,E). However in LPS challenged animals, we determined upregulation of Trx1 and TrxR in the brain of stefin B-deficient animals, when compared to WT LPS stimulated animals (Figure 1B,C,E). When we analyzed the effect of LPS challenge on control versus LPS treated WT and stefin B-deficient brain tissue, TrxR proteins were up regulated in LPS treated WT and stefin B-deficient brains, however only in stefin B-deficient brain lysates the difference after LPS challenge was statistically significant (Figure 1C,D,E).

In the brain lysates prepared from LPS challenged WT mice Trx1 was downregulated, while the reverse pattern was observed in brain lysates from stefin B-deficient mice, Trx1 was significantly upregulated in lysates prepared from LPS challenged stefin B-deficient mice, in comparison to untreated controls (Figure 1B,E). Although the levels of Prx1 were upregulated in the brain from stefin B-deficient mice, the differences between the genotypes were not statistically significant (Figure 1A). In addition, in the brain tissue of LPS treated animals, we did not detect any significant differences in Prx1 protein levels between the genotypes (Figure 1B,D). When we compared Prx1 protein levels in LPS treated WT and stefin B-deficient brain lysates, we determined down regulation of Prx1 in lysates from LPS challenged mice in both genotypes; however, the differences after LPS stimulation were significant only in brain lysates prepared from stefin B-deficient mice (Figure 1D).

### 3.2. Protein Levels of Prx1, Trx and TrxR Are Upregulated upon LPS Induced Neuroinflamamtion in Stefin B-deficient Cerebella

**Figure 2:**
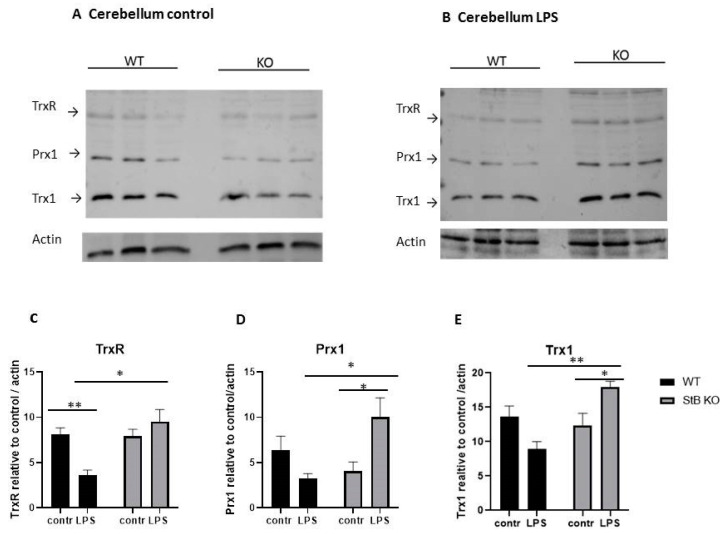
Thioredoxin reductase (TrxR), peroxiredoxin 1 (Prx1), and thioredoxin 1 (Trx1) protein levels in cerebella of stefin B-deficient (KO) and control (WT) mice after LPS challenge (n = 6). Age-matched control FVB/N mice (WT) and stefin B-deficient (KO) were left untreated or were injected with LPS (3 mg/kg body weight). 4 h after LPS challenge animals were sacrificed. Cerebellar tissue lysates of control mice (**A**) or LPS injected animals (**B**) were analyzed by Western blots with specific antibodies. Anti β-actin antibodies were used as loading controls. The levels of TrxR (**C**), Prx1 (**D**) and Trx1(**E**) in cerebellar lysates from wild type (WT) and stefin B-deficient (KO) mice in comparison to β-actin controls were quantified by ImageJ. Statistically significant differences vs. control group, * *p* < 0.05, ** *p* ≤ 0.01.

Next, we examined the protein levels of Prx1 in cerebellar tissue lysates after systemic LPS challenge in stefin B-deficient and WT mice. In addition, as in previous experiment on brain tissue lysates, we examined also Trx1 and TrxR. In the tissue lysates, form cerebella of control animals, we did not detect any significant differences between the genotypes in the protein levels of Prx1, Trx1, and TrxR (Figure 2A).

However, in LPS challenged animals, we determined upregulation of Trx1 and TrxR in the cerebellar tissue lysates from stefin B-deficient animals, when compared to the lysates from WT LPS-stimulated animals (Figure 2B,C,E). We can conclude that the upregulation of Prx1 is characteristic for stefin B-deficient cerebella following LPS-induced neuroinflammation, since in the whole brain lysates we could not detect any significant differences in the Prx1 protein between the two genotypes (Figure 1). When we looked at statistical analyses of protein levels in cerebellum of WT control animals, versus LPS challenged WT animals, the levels of TrxR, Trx1 and Prx1 were downregulated in cerebellar lysates prepared from LPS injected WT animals (Figure 2 C,D,E). In cerebellar lysates prepared from LPS challenged stefin B-deficient mice, TrxR, Trx1 and Prx1 were upregulated (Figure 2C,D,E). The differences in protein levels of Trx1 and Prx1 were statistically significant in cerebellar lysates from LPS challenged stefin B-deficient mice, when compared to untreated controls (Figure 2F).

### 3.3. Peroxiredoxin 2 (Prx2) Is Upregulated upon LPS Induced Neuroinflamamtion, but the Differences between the Genotypes Are not Significant

Prx2 is an important antioxidant enzyme in the central nervous system where it is found mostly in neurons [37,38]. Therefore, we aimed to determine the protein levels of Prx2 in LPS induced neuroinflammation in stefin B-deficient and control animals.

**Figure 3:**
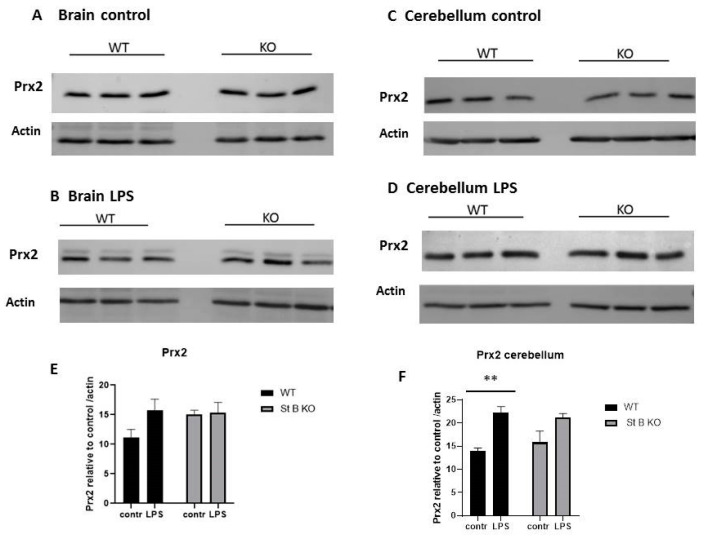
Prx2 in the brain and cerebellar tissue from stefin B deficient (KO) and control (WT) mice after LPS application. Age-matched control FVB/N mice (WT) and stefin B-deficient (KO) (n = 6) were left untreated or were injected with LPS (3 mg/kg body weight). 4 h after LPS challenge, animals were sacrificed. Brain tissue lysates (**A**) and cerebellar tissue lysates (**C**) from control mice or LPS injected animals (**B**,**D**) were analyzed by Western blots with antibodies against Prx2. Anti β-actin antibodies were used as loading controls. The levels of Prx2 in brain lysates (**E**), and Prx2 in cerebellar lysates (**F**) from wild type (WT) and stefin B-deficient (KO) mice in comparison to β-actin controls were quantified by ImageJ. Statistically significant differences vs. control group, ** *p* ≤ 0.01.

Although Prx2 protein was upregulated in stefin B-deficient brain lysates, the differences between the genotypes were not statistically significant (Figure 3A,B,E). The Prx2 protein levels in brain tissue lysates of LPS treated animals were almost identical between the genotypes (Figure 3B,E). When we compared the protein levels of Prx2 in brain lysates from control versus LPS treated animals, in WT controls Prx2 was upregulated in LPS challenged animals; however, the differences were not statistically significant (Figure 3A,B). Nor did we determine any significant differences were Prx2 protein levels in brain lysates from control and LPS challenged stefin B-deficient mice (Figure 3A,B,E). In cerebellar tissue lysates of stefin B-deficient and WT animals, we did not determine any significant differences in control, untreated animals (Figure 3C). After LPS challenge, the Prx2 protein levels were significantly upregulated in WT cerebella (Figure 3F), however, we could not determine any significant differences between the genotypes (Figure 3D,F). We concluded that Prx2 is not influenced significantly by the lack of stefin B. When we analyzed the protein levels of Prx2 in cerebellar lysates from control versus LPS treated animals, in WT controls Prx2 was significantly upregulated in LPS challenged animals (Figure 3D,F).

### 3.4. Mitochondrial Prx 3 Is Upregulated in Stefin B deficient Cerebella in Control Mice, As Well As in the Brain and Cerebella of Stefin B deficient Mice upon LPS-Induced Neuroinflamamtion

**Figure 4:**
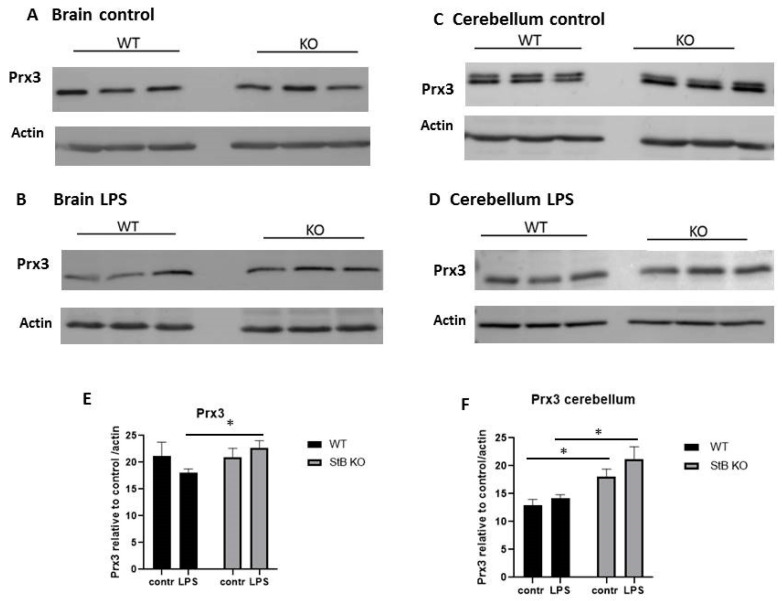
Prx3 in the brain and cerebellar tissue from stefin B deficient (KO) and control (WT) mice after LPS application. Age-matched control FVB/N mice (WT) and stefin B-deficient (KO) (n = 6) were left untreated or were injected with LPS (3 mg/kg body weight). 4 h after LPS challenge animals were sacrificed. Brain tissue lysates (**A**) and cerebellar tissue lysates (**C**) from control mice or LPS injected animals (**B**,**D**) were analyzed by Western blots with antibodies against Prx3. Anti β-actin antibodies were used as loading controls. The levels of Prx3 in brain lysates (**E**), and Prx3 in cerebellar lysates (**F**) from wild type (WT) and stefin B-deficient (KO) mice in comparison to β-actin controls were quantified by ImageJ. Statistically significant differences vs. control group, * *p* < 0.05.

Prx3 is a mitochondria-specific ROS scavenger and protects from mitochondrial damage. In our previous study we reported a marked elevation in mitochondrial Prx3 in stefin B-deficient macrophages and spleens after LPS-induced oxidative stress [36]. In this study, in control mouse brain lysates (without cerebella) we did not determine any differences in Prx3 protein levels between WT and stefin B-deficient brain lysates (Figure 4A). However, upon LPS stimulation, the protein levels of Prx3 were significantly higher in stefin B-deficient brain tissue lysates (Figure 4B,E). When we examined the protein levels of Prx3 in cerebella from WT and stefin B-deficient mice, we found significantly higher levels of Prx3 protein in the cerebellar tissue already in control, unchallenged animals (Figure 4C,F). Recently, several reports demonstrated that Prxs could be modified by acetylation, glutathionylation, phosphorylation, and thiol oxidation [39,40]. However, if double bands that we detected in Prx3 protein (Figure 4C), are a consequence of a specific post-translational modifications, it is not clear. Interestingly, in some mouse tissue double, bands of Prx3 were reported previously [41].

After LPS challenge, the Prx3 protein levels were significantly higher in brain and cerebella tissue lysates from stefin B-deficient mice than WT animals (Figure 4D,F). In addition, we analyzed the protein levels of Prx3 in brain and cerebellar lysates from control versus LPS treated animals. No significant differences in Prx3 protein in control brain (Figure 4A,B) or cerebellar (Figure 4C,D) lysates, vs. lysates from LPS treated animals were determined in WT nor in stefin B-deficient mice.

## 4. Discussion

EPM1 is a neurodegenerative disease, characterized by epileptic seizures, myoclonus, and apoptosis in cerebellum. The pathological manifestations are a consequence of the lack of expression of stefin B, an endogenous inhibitor of cysteine cathepsins. In addition, lack of stefin B was reported to be implicated in impaired redox homeostasis and increased oxidative stress-induced apoptosis [5,35]. In the mouse model of EPM1, stefin B-deficient mice, it was shown that microglial activation played an important role in the progression of the disease [6,15,42]. Moreover, neuroinflammation reported in stefin B-deficient mice was connected to inflammation in the periphery of organism. Increased amounts of pro-inflammatory macrophages, B lymphocytes in the spleen, and increased levels of pro-inflammatory cytokines and chemokines and in the serum were reported [6]. We demonstrated that upon LPS stimulation stefin B was targeted into mitochondria and prevented excessive mitochondrial ROS formation [9]. Since the signaling in macrophages and microglia could be compared, we speculated that mitochondrial ROS and activation of NLRP3 inflammasome have a crucial role in the process of neuroinflammation in EPM1 [43,44]. Interestingly, a recent study reported that differentiating neural progenitor cells from stefin B-deficient mice showed altered expression of nuclear-encoded mitochondrial genes and consequently significantly impaired mitochondrial respiratory capacity [11]. Neuroinflammation could thus promote neuronal hyperexcitability and seizures, whereas the activation of the glia cells could further contribute to the generation of seizures [45].

Herein, we evaluated the protein levels of Trx1, TrxR, and Prx1–3 in the brain and cerebella of stefin B-deficient mice. Moreover, we induced neuroinflammation with LPS and examined the levels of above-mentioned redox-sensitive proteins in the brain and cerebella, since several previous studies revealed a protective role of Prxs in LPS-induced inflammation. This included a major sensitization of Prx–deficient mice to LPS induced sepsis [32,33]. It was reported that Prx1 had a role in expression of proinflammatory cytokines in macrophages and LPS‑induced lethality was is accelerated in Prx1 deficient mice [32]. Another independent study demonstrated that the Prx2 deficient mice are more sensitive to LPS-induced sepsis than in wild-type mice [33]. Additionally, Prx3 deficient mice were reported to be more sensitive to LPS induced sepsis [34]. LPS challenge resulted in a marked elevation of Trx1 and TrxR in the brain and cerebella of stefin B-deficient mice (Figures 1,2). A recent study reported that Trx1 plays a role in inhibiting apoptosis and protects neurons from cytotoxicity through ER and mitochondria-mediated pathways [46]. The lack of stefin B was reported to be implicated in increased oxidative stress-induced apoptosis [5,35]. We propose that the increased expression of Trx1 may help to eliminate excessive ROS in stefin B-deficient cells. The increased expression of Trx1 could be a way that the organism utilizes to respond to the lack of stefin B and increased ROS formation, but it is not sufficient to prevent the pathology of disease.

Initial studies examining Prx1 and Prx2 expression in human brain revealed that Prx1 was expressed primarily in astrocytes, whereas Prx2 was expressed exclusively in neurons [38]. Later studies confirmed localization of Prx2 and Prx3 in neuronal cytoplasm, while Prx1 was found in microglia and oligodendrocytes [47]. A later study in mouse brain confirmed the neuronal localization of Prx2 and the glial expression of Prx1 [48]. Interestingly, we found Prx1 upregulated in cerebella from LPS challenged stefin B mice (Figure 2B,D), but not the brain tissue lysates (without cerebella) after LPS challenge (Figure 1A). Since it was reported that early microglial activation preceded onset of seizures and apoptosis in cerebellum in stefin B-deficient mice [16], the increased protein expression of Prx1 in cerebella of LPS stimulated stefin B-deficient mice, could reflect increased glial activation. Increased Prx3 protein in unchallenged stefin B-deficient cerebella could reflect a response to increased mitochondrial ROS generation. It was demonstrated that microglial activation is present in the brain of stefin B-deficient mice already at 2 weeks of age, later from 1 month of age, it is followed by activation of astrocytes and neuronal death [16]. Since in our experiments we used 8–12 week old mice, the pathology was already present in the brain and the upregulation of Prx3 in unchallenged cerebella may reflect the ongoing disease. Recently, not only cytosolic, but also mitochondrial localization of Prx1 was reported in yeast [49].

When we examined protein concentrations of Prx2 in brain and cerebella of LPS challenged mice, we did not detect any differences between the genotypes. It was reported that in primary glial cells, stefin B was found in progenitor and differentiated oligodendrocytes and in astrocytes, while in the cerebellum, only oligodendrocyte progenitors expressed stefin B [50]. The specific cell distribution of stefin B, or the lack of it, could reflect the differences in Prx1 and Prx2 protein expression in the tissues tested. However, we determined significant differences in protein levels of mitochondrial Prx3.

Moreover, it was reported that Prx3 deficient macrophages were more sensitive to LPS induced oxidative stress: reduced viability and increased apoptosis was reported in Prx3 deficient macrophages challenged with LPS [31]. In our previous work, we reported that the lack of stefin B resulted in the increased destabilization of the mitochondrial membrane potential and mitochondrial superoxide generation in LPS treated macrophages [9]. After LPS challenge, the Prx3 protein levels were significantly higher in brain and cerebella tissue lysates from stefin B-deficient mice, than control animals (Figure 4). Increased Prx3 protein levels could be a response to the higher generation of mitochondrial ROS in myeloid cells of stefin B-deficient mice. A result, which is in line with our published work on stefin B-deficient macrophages and spleen tissue lysates, where we determined increased mitochondrial Prx3 in stefin B-deficient animals, after LPS challenge [36].

Moreover, with experiments with stefin B-deficient mice crossed to Prx3 transgenic animals, we could determine if inflammation and pathology of the disease are ameliorated. Mitochondrial dysfunction was described as one of the essential pathological hallmarks of inflammation during epilepsy [45]. Although our study is limited to protein analysis in brain and cerebellar tissue lysates, it is the first study that examined the expression of redox-sensitive proteins in mouse model of EPM1.

## 5. Conclusions

As a conclusion, our data provide evidence that stefin B deficiency in brain results in a significant increase in the expression of the cytosolic TrxR, Trx1, and the mitochondrial Prx3, in response to LPS induced neuroinflammation. In cerebellum, not only TrxR, Trx1, and Prx3 but also Prx1 was upregulated upon LPS challenge in stefin B-deficient mice. Interestingly, Prx3 was upregulated also in unstimulated cerebella of stefin B-deficient mice. The observed results are in line with our published observation regarding protein levels Trx1, TrxR, and Prx1–3 in LPS stimulated macrophages from stefin B-deficient mice [36].

## Data Availability

The datasets used and/or analyzed during the current study are available from the corresponding authors.
